# HIPPOCAMPAL GLUTAMATE MODULATION DURING MEMORY ENCODING: ASSOCIATION WITH AGE AND SUBFIELD VOLUMES

**DOI:** 10.1093/geroni/igz038.3095

**Published:** 2019-11-08

**Authors:** Chaitali Anand, Roya Homayouni, Qijing Yu, Sruthi Ramesh, Dalal Khatib, Cheryl Dahle, Jeffrey Stanley, Naftali Raz

**Affiliations:** Wayne State University, Detroit, Michigan, United States

## Abstract

Hippocampal glutamatergic activity plays a pivotal role in memory consolidation, including the ability to form novel associations that declines with age. To test whether glutamatergic dysfunction may underpin age-related memory declines, we examined in vivo age differences in hippocampal glutamate modulation during encoding of associations, and its relationship with hippocampal subfield volumes. Proton functional magnetic resonance spectroscopy was performed on 32 young (25.1±2.8 years; 18 females) and 16 older (65.9±2.7 years; 7 females) adults to measure changes in hippocampal (randomly assigned right or left) glutamate during an object-location paired association learning task (with 12 cycles of encoding-retrieval epochs). Volumes of the dentate gyrus&CA3, CA1, subiculum, and entorhinal cortex were manually measured from T2-weighted MRI images. Memory performance differed between the age-groups [F(1, 46)=8.56, p<.01], with the older attaining a lower asymptote [t(46)=2.93, p<.05] compared to the younger. Age differences in glutamate were observed only during encoding (age-group x epoch: F(3,137)=5.28, p<.01), and varied over the epochs. Young adults showed increased glutamate during the first four encoding epochs of each cycle, with levels remaining high thereafter. Old adults evidenced a decrease in glutamate during the first four epochs, and a slow, sustained ramping-up afterwards. Including both age-groups, the maximum change in glutamate, calculated using the maximum and minimum levels during encoding, was positively associated with CA1 [F(2,39)=4.28, p<.05] and the dentate gyrus&CA3 volume [F(2,39)=4.4, p<.05], after correcting for multiple comparisons. Glutamate modulation specific to encoding may underlie age-related memory declines and be related to selected hippocampal subfield volumes.

